# Relationship between dry eye and glycosylated haemoglobin among diabetics in Ibadan, Nigeria

**DOI:** 10.11604/pamj.2019.33.14.14074

**Published:** 2019-05-08

**Authors:** Segun Isaac Olaniyan, Oluyemi Fasina, Charles Obu Bekibele, Ayobade Oluwole Ogundipe

**Affiliations:** 1 Department of Ophthalmology, University College Hospital, Ibadan, Nigeria

**Keywords:** Diabetes mellitus, dry eye, glycosylated haemoglobin, Ibadan, Nigeria

## Abstract

**Introduction:**

dry eye is a fairly common ocular surface disorder which significantly affects the quality of life of patients. This study aimed to determine the prevalence, and relationship between dry eye and glycosylated haemoglobin (HbA1c) among patients with diabetes mellitus.

**Methods:**

this was a descriptive hospital-based study conducted among patients diagnosed with diabetes mellitus and attending the Diabetic Clinic at a tertiary health facility in Ibadan, south-western Nigeria. Dry eye was assessed using the standardized Ocular Surface Disease Index Questionnaire administered to the eligible respondents on dry eye symptoms. Detailed ocular examination including the tear break-up time (TBUT) and Schirmer I test were carried out and a recent glycosylated haemoglobin value was also obtained.

**Results:**

one hundred and eighty-nine Type 2 diabetic patients were studied, with 68.8% female and a mean age of 60.2 ± 10.3 years. The frequency of dry eye among patients was 21.7% (95% CI, 15.8-27.6). The most commonly reported symptoms of dry eye were “feeling of gritty sensation” (78%, 95% CI, 65.4-90.7) and “blurred vision” (73.2%, 95% CI, 59.6-86.7) while “discomfort in windy areas” (61%, 95% CI, 46.0-75.9) was the most common environmental trigger. No statistically significant correlation was noted between dry eye and HbA1c (r = 0.086, p= 0.239), and age (r = 0.096, p = 0.1)

**Conclusion:**

dry eye is fairly common among patients with diabetes mellitus with most frequent symptoms being gritty sensation and blurred vision. No significant correlation was noted between dry eye and glycosylated haemoglobin (HbA1c).

## Introduction

Dry eye is defined as a multifactorial disease of the tears and ocular surface resulting in symptoms of discomfort, visual disturbance, and tear film instability with potential damage to the ocular surface. It is accompanied by increased osmolarity of the tear film and inflammation of the ocular surface [1]. Many patients with dry eye remain undiagnosed and untreated especially in developing countries which adversely affects their vision-related quality of life [2]. Diabetes mellitus is defined as a group of metabolic diseases characterized by hyperglycemia resulting from defects in insulin secretion, insulin action or both [3]. It is a disease of public health importance affecting 38.2 million people worldwide with half of this population living in Africa, and it is reported to be on the increase [4]. Peripheral neuropathy, nephropathy, and retinopathy are well known major complications of diabetes mellitus while other ocular complications include dry eye, cataract, glaucoma, and recurrent corneal lesions [5, 6]. Diabetes mellitus can lead to dry eye through a variety of mechanisms [7, 8] and studies [9-12] had reported a high rate of dry eye among patients with diabetes mellitus. Prevalence of dry eye in patients with diabetes has been reported to range between 27.7%-54.3% [13-16]. The mechanism of dry eye in diabetes include diabetic neuropathy, metabolic dysfunction, or lacrimal gland dysfunction [6, 17, 18]. The vision-related quality of life in patients with dry eye is affected with symptoms ranging from mild transient irritation to persistent dryness, burning, itching, redness, pain, ocular fatigue, blurred vision and reduced contrast sensitivity which often affect daily activities such as reading, watching television and driving [19-21]. There are few studies [2, 22, 23] on dry eye disease in Nigeria, but to the best knowledge of the authors, there is no study on dry eye among diabetics in the country. Dry eye can be assessed using different parameters as documented by previous authors including the tear film osmolarity, [16] symptomatology, [24, 25] use of ocular lubricants, [10] combination of symptoms and sign of dry eye, [14] and the ocular surface disease index (OSDI) score [14]. The Ocular Surface Disease Index (OSDI) has been described as a valid and reliable instrument for measuring the severity of dry eye, and its psychometric properties makes it a useful tool in clinical practice [19]. Glycosylated haemoglobin (HbA1c) reflects average plasma glucose over the previous eight to twelve weeks [26]. An international expert committee recommended that HbA1c ≥ 6.5% is diagnostic of diabetes, [27] and some complications of diabetes such as retinopathy have been shown to be predictable using HbA1c levels [28]. This study thus aims to quantify the magnitude and pattern of presentation of dry eye among patients with diabetes mellitus attending the Out-Patient Clinic of a tertiary center in Ibadan, southwestern Nigeria, and explore any correlation between HbA1c and dry eye among them.

## Methods

This was a descriptive hospital-based study carried out in the ophthalmology and diabetic clinics of a tertiary hospital in Ibadan, Nigeria between December 2014 and January 2015. A sample size of 189 was calculated based on a previously reported prevalence of dry eye among diabetics of 52%, [13] 95% confidence interval, and adjustment fornon-response rate of 10%. Consecutive patients with diabetes mellitus aged 18 years and above, attending the Diabetic Clinic of the hospital as out-patients were recruited into the study until the calculated sample size was obtained. Excluded from the study were patients having ocular conditions that could affect the definition of dry eye such as microbial conjunctivitis, those who had undergone any extraocular or intraocular surgery or manipulation, patients with other systemic diseases such as hypertension, or on medications such as antihistamines, beta-blockers, and topical eye medications and those who declined to participate in the study. Patients recruited into the study were previously diagnosed diabetics using the American Diabetes Association (ADA) Expert Panel diagnostic criteria of diabetes mellitus [3]. A four-staged design was used with an interviewer-administered questionnaire carried out in the first stage to obtain patients' demographic data, past medical history, ocular history, duration of diabetes mellitus, use of insulin and a recent HbA1c report (within one week of recruitment). The patients were then examined with the slit lamp biomicroscope for eyelids abnormalities that could interfere with the normal spread of tear film, and conjunctival disorders like pterygium. In this study, dry eye was diagnosed using the Ocular Surface Disease Index (OSDI) questionnaire which consists of 12 questions on “symptoms within the past week” and gives scores ranging from 0 (least severe) to 100 (most severe). A score of 12 was used as a cut off for normal, 13-22 for mild dry eye, 23-32 for moderate dry eye, and ≥ 33 for severe dry eye [19].

Lastly, the tear break-up time (TBUT) was done followed by Schirmer I test with topical anaesthesia 30 minutes later to avoid any interference of results. TBUT was done by instilling a drop of 2% fluorescein strip wetted with sterile water into the conjunctival sac of each eye. The time interval between the last complete blink and the appearance of a random dark spot on the cornea under the cobalt blue filter of the slit-lamp was recorded with a stopwatch, and the mean of three timings was noted. A value of 10 seconds or less was considered as abnormal [29]. Schirmer I test was done with patients seated in a darkened room with fans and air-conditioning switched off. A pre-calibrated Schirmer strip (Whatmann filter paper no. 41) was placed at the inferior conjunctival sac at the junction of the lateral third and medial two thirds. Participants were asked to look straight and allowed to blink. After 5 minutes, the test strips were removed and the amount of wetting of the strips was recorded with a value less than or equal to 5mm considered as abnormal. For each study participant, Schirmer I test and TBUT were carried out in both eyes and the eye with the worse result was included in the analysis [29]. Ethical approval was obtained from the hospital’s ethical committee. Written and informed consent was obtained from the patients and the study followed the tenets of the Helsinki declaration.

**Data management and analysis:** data collected was analyzed using the Statistical Package for Social Sciences (SPSS) software (SPSS for windows version 21.0; SPSS Inc, Chicago, Illinois). Summary statistics were presented using frequency tables, charts, means and rates. Chi-square and Fishers exact tests were used for categorical variables. Spearman rank-order correlation co-efficient was used to determine the relationship between dry eye and HbA1c. Level of statistical significance was set at < 5%.

## Results

**Figure 1:**
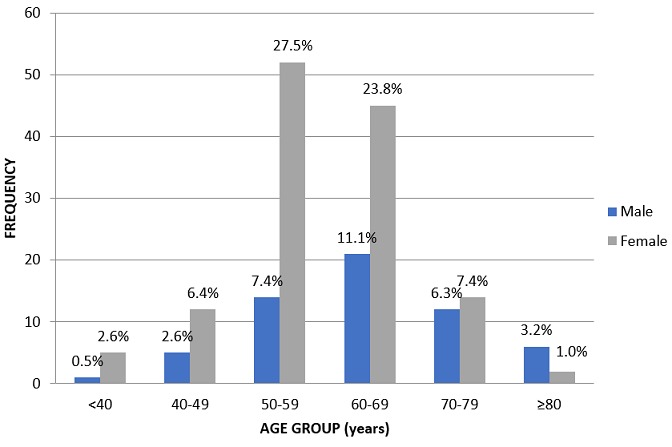
age and gender distribution of patients

One hundred and eighty-nine patients participated in the study of which 59(31.2%) were males, (Figure 1) (M: F = 1: 2.2) and the mean age was 60.2 ± 10.3 years. The mean age for the males was 63.5 ± 11.3 years and 58.7 ± 9.4 years for the females. All the patients in the study had type 2 diabetes mellitus with a mean duration of 9.8 ± 7.3 years (range, 6 months to 37 years) (Table 1). One hundred and thirty (68.8%) patients were on oral medications only while 22(11.6%) were on treatment with insulin (Table 1). Glycosylated haemoglobin < 6.5% was observed in 106(56.1%) patients while ≥ 9% was noted in 25(13.2%) patients. The mean value for HbA1c was 7.1% (Table 1). The most common symptoms of dry eye among all the patients was “gritty sensation” (49.7%) and “blurred vision” (31.2%), while the most common environmental trigger was “discomfort in windy conditions” (21.2%), and the least common was “discomfort in low humidity and in air-conditioned areas” (0.5%). These symptoms were not exclusive as some patients experienced more than one symptom (Table 2). The prevalence of dry eye in this study was 21.7% (95% CI, 15.8-27.6) and this was higher among male patients (25.5%, 95% CI, 14.3-36.5) than females (20.0%, 95% CI,13.1-26.9) (p = 0.267), with 143(78.3%) patients classified as normal (Table 3). There was an increase in the prevalence of dry eye with increasing age (p= 0.107) as well as with specific age group (Table 4). There was no significant association between dry eye and some characteristics including the tear break-up time, Schirmer's test, and Hb A1c among the patients with Chi square analysis (Table 5). Also, there was no significant correlation between dry eye and glycosylated haemoglobin (r = 0.086, p = 0.239), dry eye and age (r = 0.096, p = 0.190), and, dry eye and duration of diabetes mellitus (r = 0.027, p = 0.714) among the patients.

**Table 1:** duration of diabetes mellitus, type of treatment and glycosylated haemoglobin (HbA1c) values the among patients

Duration of diabetes mellitus	No. of patients	Percentage (%)
0- 6months	2	1.1
> 6months-1year	5	2.6
> 1year-5years	60	31.7
> 5years-10years	40	21.2
> 10years-20years	65	34.4
> 20years	17	9.0
Total	189	100
Treatment type		
Oral medication	130	68.8
Insulin	22	11.6
Oral medication + Insulin	34	18.0
Diet only	3	1.6
Total	189	100
HbA1c value		
Less than 6.5%	106	56.1
6.5% to less than 9.0%	58	30.7
Greater than 9.0%	25	13.2
Total	189	100

**Table 2:** frequency of symptoms among patients with dry eyes using the OSDI score

Dry eye symptoms (n=41)	Frequency	Percentage (%)	95% C.I
Gritty sensation	32	78.0	65.38 - 90.71
Blurred vision	30	73.2	59.60 - 86.73
Discomfort in Windy conditions	25	61.0	46.04 - 75.90
Light sensitivity	17	41.5	26.38 - 56.53
Painful eyes	12	29.3	15.34 - 43.19
Poor vision	12	29.3	15.34 - 43.19
Limitation in Driving	10	24.4	11.24 - 37.53
Limitation in Reading	9	22.0	9.28 - 34.62
Limitation in Watching TV	6	14.6	3.81 - 25.45
Discomfort in Low humidity areas	1	2.4	-0.22 - 7.16
Limitation in Computer/ ATM use	1	2.4	-0.22 - 7.16
Discomfort in Air conditioned areas	1	2.4	-0.22 - 7.16

**Table 3:** prevalence and grades of dry eye among the patients using the Ocular Surface Disease Index Score

OSDI	Dry eye disease grade	No. of patients	Percentage (%)
0-12	Normal	148	78.3
13-22	Mild	33	17.5
23-32	Moderate	4	2.1
33-100	Severe	4	2.1
**Total**		189	100

**Table 4:** prevalence of dry eye by age group

Age group (years)	No. of patients	No. with dry eye (%)	% of total
<40	6	0 (0.0)	0.0
40-49	17	2 (11.8)	1.1
50-59	66	15 (22.7)	7.9
60-69	66	16 (24.2)	8.4
70-79	26	6 (23.1)	3.2
≥80	8	2 (25.0)	1.1
**Total**	189	41	21.7

**Table 5:** association between dry eye (using the OSDI score) and some characteristics in patients with diabetes mellitus

Characteristics	Dry eye		Pearson Chi-square	P-value
	Present N (%)	Absent N (%)		
**Age (years)**				0.415
34-59	17(19.1%)	72(80.9%)	0.665	
≥ 60	24(24.0%)	76(76.0%)		
**Gender**				0.402
Male	15(25.4%)	44(74.6%)	0.703	
Female	26(20.0%)	104(80%)		
**Years of diabetes**				0.321
≤ 10	26(24.3%)	81(75.5%)	0.986	
˃ 10	15(18.3%)	67(81.7%)		
**Insulin use**				0.1367
Yes	16(28.6%)	40(71.4%)	2.214	
No	25(18.8%)	108(81.2%)		
**HbA1c value (%)**				0.287
< 6.5	20(18.9%)	86(81.1%)	1.134	
≥ 6.5	21(25.3%)	62(74.7%)		
**Tear break up time**				0.316
≤ 10s	36(23.1%)	120(76.9%)	1.007*	
˃ 10s	5(15.2%)	28(84.8%)		
**Schirmer’s test**				0.554
≤ 5mm	10(20.8%)	38(79.2%)	0.350	
˃ 5mm	31(22.0%)	110(78.0%)		

## Discussion

The prevalence of dry eye in this study was 21.7% (95% CI,15.8-27.6), similar to findings of Kaiserman *et al.* [10] (20.6%) and the Beaver Dam study [24, 25] (18.1%). Fuerst *et al.* [13] however, reported a prevalence of 52%, and this may be attribuited to the longer duration of diabetes (mean duration, 11.4 years [13] vs 9.8 years, present study) and racial differences among the studied participants. Longer duration of diabetes mellitus has been documented to correlate with increase in the prevalence of dry eye among the patients [14]. Dry eye was more common among males (25.5%, 95% C.I, 14.3-36.5) than females (20.0%, 95% C.I,13.1-26.9) but this was not statistically significant. Kaisermann *et al.* [10] and Fuerst *et al.* [13] also noted no significant difference in dry eye symptoms between sexes, although higher frequencies were found in females. Studies [24, 30] have shown that dry eye is more common among females in the normal population because of hormonal changes associated with menopause, however, in diabetics no gender predilection for dry eye has been observed and it was postulated that the association between female gender and dry eye is neutralised in the patients by the disease [10]. The prevalence of dry eye increased with age in this study, though, the association was not statistically significant (p = 0.415). This is similar to previous reports, [10, 13, 16] and has been attributed to the reduction in tear flow and volume, increased osmolarity, decreased tear film stability as well as alteration in the meibomian lipid composition of tears [31] with age. The duration of diabetes mellitus did not correlate with dry eye in this study (p = 0.714) similar to findings by Najafi *et al.* [16] but in contrast with the findings of Manaviat *et al.* [14]. Microvascular damage of the lacrimal gland with impairment of lacrimal gland function that has been implicated in the aetiopathogenesis of dry eye is known to correlate positively with a longer duration of diabetes mellitus [28]. Fuerst *et al*. [13] on the other hand, reported fewer dry eye symptoms among patients with longer duration of diabetes which they attributed to a possible reduction in corneal sensation. There was no statistically significant correlation between dry eye and HbA1c level in this study (p = 0.239). This is similar to reports of Fuerst *et al.* [13] and Sagdik *et al.* [32] but in contrast to findings in some studies [10, 12, 14, 16] where HbA1c had a significant positive correlation with dry eye. The overall fair glycemic control in our patients (mean HbA1c = 7.0 %) might have accounted for the lack of correlation in this study. Poor glycemic control is associated with microvascular complications of the lacrimal gland which impair lacrimal gland function causing dry eye among diabetics [28].

Treatment of diabetes with insulin was not statistically associated with dry eye in this study, (p = 0.1367). This is similar to previous studies [12, 13, 16] which reported no association between the type of treatment for diabetes and dry eye, thus, suggesting that insulin therapy does not affect the severity of dry eye among patients with diabetes mellitus. The most common symptom of dry eye was “gritty sensation”, followed by “blurred vision” and “discomfort in windy conditions”, similar to the study by Manaviat *et al.* [14]. These symptoms resulted from the disturbance in the quantity and quality of the pre-corneal tear film resulting in ocular surface inflammation [19, 33-35]. Schirmer test score was abnormal in 20.8% of patients with dry eye while tear break up time was abnormal in 23.1% with neither test having any significant association with dry eye symptoms. Manaviat *et al.* [14] reported 11.5% of their patients had both abnormal tear break up time and Schirmer's test score with no significant association with subjective symptoms of dry eye. The lack of association between symptoms and signs of dry eye among patients with the disease has been documented [36]. However, decreased basal tear secretion indicated by abnormal Schirmer's test score has been reported in patients with diabetes mellitus, [6, 17] and this had been attributed to the microvasculature damage of the lacrimal glands and autonomic neuropathy leading to lacrimal gland dysfunction [37]. Limitations to this study include the absence of patients with type 1 diabetes mellitus such that associations between dry eye and type 1 diabetes mellitus could not be assessed. More studies involving control groups will be helpful in evaluating further the relationship between dry eye and diabetes.

## Conclusion

In conclusion, dry eye is fairly common among patients with type 2 diabetes mellitus in our black African population with most of the affected patients experiencing the mild form of the disease. No significant correlation was noted between dry eye and glycosylated haemoglobin (HbA1c).

### What is known about this topic

- Dry eye affects the ocular surface and results in tear film instability;
- Prevalence of dry eye increases with age and is higher among females;
- Patients with diabetes mellitus have higher prevalence of dry eye disease.

### What this study adds

- A prevalence value for dry eye was derived for the region which can be used in further studies;
- No significant gender predilection for dry eye was noted in this study;
- No significant correlation was also noted between dry eye and glycosylated hemoglobin.
